# Ranking the Healthcare Resource Factors for Public Satisfaction with Health System in China—Based on the Grey Relational Analysis Models

**DOI:** 10.3390/ijerph18030995

**Published:** 2021-01-23

**Authors:** Xinxin Peng, Xiaolei Tang, Yijun Chen, Jinghua Zhang

**Affiliations:** 1 School of Business, Macau University of Science and Technology, Macao 999078, China; 1809853jbm30001@student.must.edu.mo (X.P.); xltang@must.edu.mo (X.T.); 1909853mbc20001@student.must.edu.mo (Y.C.); 2 School of Business, Jiangsu University of Technology, Changzhou 213000, China

**Keywords:** healthcare resources, public satisfaction with the health system, GRA models

## Abstract

(1) Background: Public satisfaction with the health system is a very important comprehensive indicator. Given the limited healthcare resources in a society, it is always important for policymakers to have full information about the priority and the ranking order of the factors of healthcare resources for improving public satisfaction. (2) Methods: Grey Relational Analysis (GRA) is advantageous for satisfaction analysis because satisfaction is a “grey concept” of “having a clear boundary but vague connotation”. The data were from the CGSS and the China Health Statistics Yearbook (2013 and 2015), with a total of 15,969 samples (average satisfaction score = 68.5, age = 51.9, female = 49.4%). (3) Results: The government’s percentage of total expenditure on healthcare was ranked as the most important factor for public satisfaction with the health system in China in both 2013 and 2015. The second most important factor changed from “Out-of-pocket percentage of individuals” in 2013 to “Hospital beds per thousand populations” in 2015. Meanwhile, “Healthcare workforce per thousand populations” increased from the least important factor in 2013 to the 3rd in 2015. Disparities in the ranking orders of the factors among regions of China were identified too. (4) Conclusions: The analysis results suggest that during recent years the priority of Chinese residents’ healthcare satisfaction for healthcare resources has shifted on the national level from economic affordability to more intensive “people-centered” services, while the regional disparities and gaps need to receive more attention and be further improved in the healthcare reform of next round.

## 1. Introduction

### 1.1. Healthcare Satisfaction

The public satisfaction of the health system is the public’s subjective perception and evaluation of the health system in a nation [1], reflecting the difference between the public expectation and the actual performance of the health system [2].

The investments of health resources, such as total health expenditure as a percentage of GDP or government expenditure as a percentage of total health expenditure, may affect the public satisfaction of the health system according to its performance [3,4,5]. For example, unaffordable health care and poor access are important factors for explaining why public healthcare satisfaction in the US is generally lower than that of other developed countries [6].

Public healthcare satisfaction is a mixture of personal experiences with the healthcare system, beyond the provision of quality services [7,8]. It also varies according to demographic and socioeconomic characteristics, health status, health insurance status, and individual ideology [9,10,11,12,13], and is influenced by the social welfare culture and media portrayals of the healthcare system in a country [3,14].

Apart from dimensions of safety, effectiveness, responsiveness, affordability, accessibility, and patient-centeredness [15,16,17,18,19,20], public satisfaction is also a very important comprehensive indicator of the health system [1,6,14], because it includes not only users (the patients) but also non-users (the healthy population) in the system [7]. Hence, it can provide meaningful information (such as key factors of public satisfaction improvement) for health policy reform [21,22] and government’s decisions making (such as fiscal expenditure allocation and healthcare service supplies) [23,24,25,26]. Since governments in modern societies usually play key roles in the health systems [6,14], the public’s satisfaction with the health system is closely associated with the public satisfaction with and public trust in governments [23].

Understanding the priority of public satisfaction of the health system is important. Efficiency and effectiveness with limited health resources in a healthcare system are extremely important and even the smallest failures cannot be tolerated [27]. Meanwhile, the priority of public satisfaction differs in each health system, and policymakers need to determine priorities of policies or reforms in their own countries [6,8,14,22,27,28,29].

### 1.2. China’s Health System

The health system in China is a public hospital-based delivery system, which mainly includes primary, secondary, and tertiary services, providing healthcare services separately [5,7]. The tertiary hospitals are usually considered to have high-quality services [30,31,32,33,34] and are in high demand by large volumes of patients, who are free to self-refer to preferred hospitals due to the lack of a “gatekeeper” system in the primary healthcare institutions of China [35]. Consequently, all tertiary hospitals face the challenges of long waiting times and a shortage of beds [36].

The healthcare expenditure in China consists of three parts: The government, the insurance company, and individual Out-of-Pocket (OOP) payment [37]. More than 95% of the Chinese population has a least one type of health insurance coverage [36,38].

With the rapid growth of the economy in China, the annual disposable income per capita in China increased from RMB 18,301.8 (about USD 2614 (the exchange rate between the US dollar and the Chinese RMB in this paper is about 7 RMB per dollar)) in 2013 to RMB 21,966.2 (about USD 3138) in 2015 (China Statistical Yearbook 2016: p168). The demand for adequate and quality healthcare services has increased rapidly nationwide and the shortage has reduced public satisfaction in China [38,39]. At the beginning of the 21st century, “seeing a doctor is hard, seeing a doctor is expensive” became the public expression of dissatisfaction with the Chinese health system [38]. In some extreme cases dissatisfactions even fermented into violent attacks on physicians [35,38,40].

Taking the above-mentioned factors into full consideration, the government in China emphasized the importance of public satisfaction with the health system and has set it as one of the main goals of China’s ongoing health policy reform [38,41].

While being improved during recent years, there are socioeconomic and geographical disparities in healthcare source allocation in China due to uneven regional economic growth and inconsistent healthcare development [30,42]. According to the official classification standard in China, the provinces in China can be grouped into one of the economic regions (East, Central, West, and Northeast regions). East China is the most developed region in China. As a general phenomenon, from the east to the central and the west in China, the economic development level gradually becomes lower. Additionally, there are four municipalities (Beijing, Shanghai, Tianjin, and Chongqing), which are megacities with abundant healthcare resources. During 2013 and 2015, Beijing had the highest “per capita Gross Domestic Product” (about USD 14,666), and the West China region had the lowest level (about USD 5115). Shanghai had the highest “per capita Disposable Income of Households” (about USD 6689) and West China had the lowest (about USD 2201) (China Statistical Yearbook 2013–2015, and the exchange rate between the US dollar and RMB is about 7 RMB per dollar).

Currently, most of the existing literature focuses on China’s healthcare reform [35,36,38,39,41,43,44], while a small volume of literature examines the public satisfaction with the health system [45,46], from the perspective of primary healthcare [47], and basic health insurance respectively [48], but few about the ranking or priority order of the factors related [28].

### 1.3. Aims of this Study

This study intends to rank key factors of healthcare resources that affect public satisfaction within the health system of China [28]. Applying the Grey Relational Analysis (GRA) models, this paper aims (1) to rank the factors of healthcare resources by their relationship with the public satisfaction in China from 2013 to 2015; (2) to examine how the ranking order of the factors of healthcare resources has changed over the years; (3) to explore how the rankings of these factors vary among different regions in China during 2013 and 2015.

## 2. Research Methods

### 2.1. Grey System Theory and Grey Relational Analysis (GRA) Models

Grey system theory is based on the observation and the fact that all-natural and social systems are uncertain systems, which contain various types of uncertainties and noises due to disturbances from internal or external sources, or due to the limitations of human knowledge and cognition. The fundamental characteristics of uncertain systems are the incompleteness in the information of the system or the data available [49]. Incomplete information in the system may involve the elements (parameters), the structure, the boundaries, and the behaviors of a system. Meanwhile, incomplete information of data is demonstrated by the inaccuracies of data and can be categorized into three types according to the original sources, namely, conceptual, level of perspective, and prediction inaccuracies. For instance, the frequently used concepts, such as “large”, “small”, “fat”, “thin”, “good”, “bad”, “young”, and “beautiful”, are inaccurate due to subjectivity in these concepts and lack of comprehensive definition [49].

The fundamental meaning of “grey” is the incompleteness in the information. Since the information quantity and quality of a system form a continuum from a total lack of information to complete information, grey system theory uses “white” to represent completely known information, “black” for unknown information, and “grey” for partially known and partially unknown information [49]. A system of incomplete information is defined as a grey system accordingly [49].

Grey system theory has further developed a series of models to analyze and make use of the information contained in a grey system. Grey Relational Analysis (GRA) (also called Deng’s Grey Incidence Analysis model) is one of the most widely used models of grey system theory, which was initially proposed by Professor Deng Julong [27,49,50,51,52]. GRA utilizes the degree of similarity of geometric curves of available data sequences to determine whether they are closely associated or not. The more similar the curves are, the more relevant the sequences are, and vice versa [27,49]. GRA models include various types of “grey relational degree” numbers (or “degree of grey incidence”). A variation of the models is briefly explained as follows.

#### 2.1.1. Deng’s GRA Model

Depending on the correlation coefficient of specific points, Deng’s GRA model reflects the inter-influences among the factors analyzed [49,51,53], hence, it has been widely adopted in various fields of research [52,54,55,56,57,58]. According to Liu et al. [49], Deng’s GRA model follows the computing steps as described below.

Step 1, the dependent variable forms the reference sequence *x_0_* and the independent variables form the comparison sequence *x_i_* (*i* = 1, 2, 3…, *n*) [53].

Step 2, for observation time or observation number *k* (*k* = 1, 2…, *m*), grey relational coefficient (also called “grey incidence coefficient, or point relation coefficients), γ*_i_* *(k),* is calculated according to Equations (1)–(3):

(1) $$\gamma_{i}\left(x_{0}\left(k\right),x_{i}\left(k\right)\right)=\frac{\min\min|{x_{0}}^{\prime }\left(k\right)-{x_{i}}^{\prime }\left(k\right)|+\varepsilon \max\max|{x_{0}}^{\prime }\left(k\right)-{x_{i}}^{\prime }\left(k\right)|}{\left|{x_{0}}^{\prime }\left(k\right)-{x_{i}}^{\prime }\left(k\right)|+\varepsilon \max\max|{x_{0}}^{\prime }\left(k\right)-{x_{i}}^{\prime }\left(k\right)\right|}$$

(2) $$\mathrm{where}\;\;\;\;\;\;\;\;\;\;\;\;\;\;\;\;\;\;\;\;\;\;\;\;\;\;\;\;\;\;\;\;x_{i}^{\prime }\left(k\right)=\frac{x_{i}\left(k\right)}{\bar{x_{i}}\left(k\right)},$$

(3) $$\bar{x_{i}}\left(k\right)=\frac{1}{n}\sum_{k=1}^{n}x_{i}\left(k\right)\;\;\;\;\;\;\;\;\;\;\;\;\;\;\;\;\;\;\;\;$$

*i* = 1, 2…, *n*;
*k* = 1, 2…, *m*; (*k* indicates observation time, or observation number);
ε is called the resolution coefficient, which has a range of value from 0 to 1, and often takes the value of 0.5 even though the rationale behind this assumption is debatable [27,28].

Step 3, based on the results of γ*_i_* *(k)* from Step 2, grey relational degree (also called Deng’s degree of grey incidence,) *β_i_(k)* is calculated according to Equation (4):

(4) $$\beta_{i}\left(k\right)=\frac{1}{n}\sum_{k=1}^{N}\gamma_{i}\left(k\right).$$

Step 4, rank the grey relational degree *β_i_(k)* according to the numerical values. The higher the correlation degree is, the higher the ranking is [49].

Apart from Deng’s GRA model, there are new variants and developments in GRA, such as Absolute GRA, Relative GRA, the first synthetic GRA (SDGRA), and the second synthetic GRA (SSGRA).

#### 2.1.2. Absolute GRA

Derived from Deng’s GRA, the Absolute GRA utilizes the specific point grey and analyzes the correlations between the factors [28,49]. Yu et al. [59] and Tung and Lee [60] highlights the deployment of an absolute GRA model for measuring the association/correlation between variables/parameters as:

(5) $$\varepsilon_{0i}=\frac{1+\left|s_{0}\right|+\left|s_{i}\right|}{1+\left|s_{0}\right|+\left|s_{i}\right|+\left|s_{0}-s_{i}\right|}\;\;\;\;\;$$

where

(6) $$s_{0}=\overset{n-1}{\underset{k=2}{\sum }}x_{0}^{0}(k)+\frac{1}{2}x_{0}^{0}\left(n\right),s_{i}=\overset{n-1}{\underset{k=2}{\sum }}x_{i}^{0}(k)+\frac{1}{2}x_{i}^{0}\left(n\right)$$

and

(7) $${\;X}_{i}^{0}=\left\{\;x_{i}^{0}\left(1\right),\;x_{i}^{0}\left(2\right)\ldots ,x_{i}^{0}\left(n\right)\right\}$$

where *i* and *k* are the same as above.

#### 2.1.3. Relative GRA

Further, the Relative GRA utilizes the integral visual angle [49,59]. The original sequence is zeroed, and the mean value is taken as the initial point. Relative GRA would be given by [49]:

(8) $$\gamma_{0i}=\frac{1+\left|{s_{0}}^{\prime }\right|+\left|{s_{i}}^{\prime }\right|}{1+\left|{s_{0}}^{\prime }\right|+\left|{s_{i}}^{\prime }\right|+\left|{s_{0}}^{\prime }-{s_{i}}^{\prime }\right|}$$

where

(9) $${s_{0}}^{\prime }=\overset{n-1}{\underset{k=2}{\sum }}{x_{0}^{0}}^{\prime }(k)+\frac{1}{2}{x_{0}^{0}}^{\prime }\left(n\right),{s_{i}}^{\prime }=\overset{n-1}{\underset{k=2}{\sum }}{x_{i}^{0}}^{\prime }(k)+\frac{1}{2}{x_{i}^{0}}^{\prime }\left(n\right),$$

and

(10) $${X_{i}^{0}}^{\prime }=\frac{X_{i}}{x_{i}\left(1\right)}=\left\{\;{x_{i}^{0}}^{\prime }\left(1\right),\;{x_{i}^{0}}^{\prime }\left(2\right)\ldots ,{x_{i}^{0}}^{\prime }\left(n\right)\right\},$$

where $x_{i}^{0}{}^{\prime }\left(n\right)$ is the initial zero of *x_i_* (*n*), meanwhile *i* and *k* are the same as above.

#### 2.1.4. SDGRA and SSGRA

The SDGRA model evolved from Absolute GRA and Relative GRA [49]. It has been further developed to reflect the line of similar degree and the relative to the proximity of the pilot’s rate of change [49]. It is a comprehensive characterization of whether a sequence has a closer connection between several indicators [28].

The SSGRA model has incorporated the advantages of both Deng’s GRA model and the Absolute GRA model [27,28], and “reflects overall closeness between two sequences based on particular points and integral perspectives” [28].

Both SDGRA and SSGRA models can be expressed by the following equation, generally, θ = 0.5 [27,28].

(11) $$\rho_{ij}=\theta \varepsilon_{ij}+\left(1-\theta \right)\gamma_{ij},\theta \epsilon \left[0,1\right]\;$$

The detailed computing steps to calculate the GRA models discussed above are reported in Javed et al. [27,28] and Liu et al. [49].

### 2.2. Application and Advantages of GRA Models

The grey features of public satisfaction meet the basic requirement of Grey Relational Analysis (GRA) [27,49]. Because satisfaction actually is a “grey concept”, which has features of “having a clear boundary but vague connotation” [28,49,50,52], despite that literature in existence often simply treat an individual’s satisfaction as a dichotomous variable taking the value of 1 (satisfied) and 0 (dissatisfied) [3,21,61].

Compared with the regression models, GRA methods have several advantages in providing information for decision making [62]. Firstly, unlike models based on statistics and probability theory, GRA models do not need a typical probability distribution nor a large sample size [27,29,49]. Secondly, under many decision or policy situations, it is often more reasonable to describe the correlations among factors, or the order of the relationships, with Grey Relation Degree, rather than with specific numerical values of some estimated coefficients [52,53,63].

GRA models have been successfully applied in many fields, such as engineering [55], environmental science [56], and management [57]. For healthcare management studies, GRA models have been applied to study patient satisfaction [27,29,64], healthcare service quality [28,64,65], performance [62], efficiency [66,67], healthcare resource allocations [68], etc.

This study adopted variations of GRA models to perform the analysis. For the dynamic relationship among the factors, the centralized trend (e.g., the average) would be used by Deng’s GRA model, in which the arithmetic mean is taken as the initial point, and the order of data does not affect the operation of those GRA models [53]. The full sample of both 2013 and 2015 was first analyzed. Following the same pattern, the data of each year of 2013 and 2015 were analyzed separately to identify changes over time. Besides, the subsamples of each municipality or region in China were examined respectively for the regional differences.

The Gray Level Correlation Software 7.0.1 (http://igss.nuaa.edu.cn/) developed by Nanjing University of Aeronautics and Astronautics was used to process Deng’s/Absolute/Relative/SDGRA model data. Microsoft Excel software was used to calculate SSGRA. STATA15 is used for obtaining descriptive statistics of the raw data.

## 3. Data Sources

The survey data analyzed in this study is from the Chinese General Social Survey (CGSS) 2013 and 2015, which performed national surveys regarding the Chinese citizens’ quality of life in two waves during 2013 and 2015. The CGSS was the first nationally representative survey in mainland China. Data and multi-stage stratified sampling design covered 31 Chinese provinces and cities. Its main sampling unit (PSU) is a county-level unit. There are 2762 PSUs in the sampling frame. In each wave of surveys, CGSS took 12,000 family samples and randomly selected an adult interviewee (18 years or older) from each family to conduct face-to-face family interviews by using the KISH grid program [7]. Sampling weights include general population parameters that reflect the survey year.

The public healthcare resource data are on the province level and obtained directly from the China Public Health Statistical Yearbook 2013 and 2015.

## 4. Variables

### 4.1. Dependent Variable

A satisfaction score with the public health system in China, evaluated by each respondent of the CGSS survey (2013 and 2015). A survey question asked, “Comprehensively considering all aspects how is your overall satisfaction with healthcare services?” Respondents need to score the degree of their satisfaction based on their subjective evaluation, ranging from 0–100 points, where “0” means completely dissatisfied and “100” means completely satisfied.

Apart from the satisfaction score of the total sample, the average score of each major economic region or municipality in China was also calculated.

### 4.2. Explanatory Variable

Explanatory variables include the total health expenditure as a percentage of GDP [3], the governments’ percentage of the total health expenditure on healthcare [3,4,5], out-of-pocket percentage of individuals [6,13,37], representing general supply and affordability of healthcare service [15,16,17,18,19,20,69].

The density of hospital beds (per thousand population) partly represents the state of hospital infrastructure [15] and measures the accessibility of healthcare [15,16].

The density of the health workforce (per thousand population) reflects the accessibility and the responsiveness of healthcare [69].

## 5. Analysis and Results

### 5.1. Descriptive Statistics

As shown in Column (1) of Table 1, the average satisfaction score of the total sample of 15,969 surveys (5566 in 2013 and 10,403 in 2015 separately) is 68.5 points (out of 100 points). Specifically, the average satisfaction score increased from 66.21 in 2013 to 69.73 in 2015. Among the regions and municipalities, Chongqing (73.94) had the highest satisfaction score, followed by Central China (70.53) and West China (70.49) respectively. The lowest satisfaction was in Northeast China (63.88), followed by Shanghai (64.33).

In Column (4) of Table 1, the “Total health expenditure as a percentage of GDP” is highest in Beijing, which has the most advanced medical service as the capital and has the highest ratio of government employees among its residents. The high ratio in West China region can be explained by the relative relationship between the increased healthcare spending and the lower GDP level in this region. Results in Column (5) and (6) of Table 1 indicate that the economic burdens of healthcare are below the average level in municipalities (Beijing, Shanghai, and Tianjin) and East China (the most developed region in China), whereas Central and Northeast China are on the high end. As shown in Columns (7) and (8), healthcare resources measured in terms of hospital beds and workforce are highly concentrated in Beijing and Shanghai.

### 5.2. Results of the GRA Models

The GRA results of the full sample are shown in Table 2. “Government’s percentage of total expenditure on healthcare” is consistently estimated to have the highest correlation to public healthcare satisfaction in all models. Meanwhile, the ranking orders of the other four factors are not consistent when different GRA models are applied.

As shown in Table 3, for 2013 “Government’s percentage of total expenditure on healthcare” and the “Out-of-pocket percentage of individuals” are consistently ranked as the top two factors by all models, except for the Relative GRA model.

As shown in Table 4, for 2015 “Government’s percentage of total expenditure on healthcare” is ranked in 1st place consistently by all models of GRA. In both the SDGRA and the SSGRA models, the order of the other four factors is the same, but the specific ranking changed in 2015. “Hospital beds per thousand population” ranked 2nd, then “Healthcare workforce per thousand population”, “Total health expenditure as a percentage of GDP”, and “Out-of-pocket percentage of individuals”.

Table 5 reports the GRA results in eight regions of China respectively. For simplicity, only results by Deng’s GRA model are reported here because it is the classic model and most widely used, and results by other models are largely consistent. “Government’s percentage of total expenditure on healthcare” is estimated to have the highest correlation to public healthcare satisfaction in Northeast China, Beijing, and Chongqing. “Total health expenditure as a percentage of GDP” is ranked in first place in both Central China and Shanghai. The most important factors affecting public healthcare satisfaction are different in East China, West China, and Tianjin. They are “Hospital beds per thousand population”, “Out-of-pocket percentage of individuals”, and “Healthcare workforce per thousand population” respectively.

## 6. Discussion

Combining a survey sample of 15,969 respondents and the relevant healthcare resources data between 2013 and 2015 from across China, this study applied a set of GRA models to rank the healthcare resource factors for public satisfaction with the health system in China. The GRA models consistently rank “Government’s percentage of total expenditure on healthcare” for the 1st place. Indeed, government health expenditure in China has increased by 30.69% from RMB 954.58 billion (about USD 136.37 billion) in 2013 to RMB 1247.53 billion (about USD 178.22 billion) in 2015 (China Public Health Statistical Yearbook 2018: p93), helping to increase the overall healthcare supply [70] and to reduce the financial burden of healthcare expenses on citizens [14,45].

The GRA results indicate that the ranking order of the “Hospital beds per thousand population” rose from 3rd place in 2013 to 2nd place in 2015. Similarly, “Healthcare workforce per thousand population” rose to 3rd place in 2015 in both the SDGRA and the SSGRA models. These changes in the ranking order of the factors suggest that, after the primary care availability, the residents in China demand more people-centered intensive care. Hospital bed density in China has increased from 5.55 per thousand populations in the year 2013 to 6.31 per thousand population in the year 2015, indicating improved access and availability of healthcare [15]. Meanwhile, the density of the healthcare workforce in China has increased from 2.74 per thousand populations in 2013 to 2.89 per thousand populations in 2015. For the next stage of health system reform in China, more resources need to be directed toward the healthcare workforce [42].

Finally, GRA results indicate significant regional disparities regarding the ranking orders of the healthcare resource factors. In East China (the most economically developed region in China), “Hospital beds per thousand populations” is ranked for 1st place. While in West China (the least economically developed region in China), “Out-of-pocket percentage of individuals” is ranked as the most important factor, indicating the importance of affordability of health services. Having leading positions among the municipalities, “Government’s percentage of total expenditure on healthcare” is ranked 1st place in Beijing and Chongqing, and 2nd place in Shanghai and Tianjin. These results are consistent with the heavy government investment in the major public hospitals in these most important cities in China.

## 7. Conclusions

The findings of this study based on GRA models suggest that the local governments in China need to continue to maintain the overall level of healthcare service expenditure to meet the needs of the residents, despite the extra-economic burdens and costs occurring during the COVID-19 pandemic. Since the satisfaction with healthcare is shifting on the national level from economic affordability to people-centered care, the next stage of healthcare reform in China needs to channel more resources towards increasing quality service volume. Besides, customized strategies are recommended to address regional disparities and gaps in the priorities of healthcare resources.

### Limitations of this Study

The GRA models have some limitations. Deng’s GRA model assumes weights of criteria are equally distributed, but this assumption may not hold in the real world [28,49]. Also, different methods of mean adopted by the GRA models may lead to different results [29].

Secondly, the satisfaction score of residents in this study is from self-reported survey data. Its measurement is influenced by the subjective evaluation of respondents [7,71].

Additionally, this study is limited by data availability of healthcare resources, which are aggregated on the provincial level. The ranking orders can’t directly capture the gaps between urban and rural areas, especially poverty-stricken areas.

## Figures and Tables

**Table 1:** Descriptive statistics of the satisfaction scores in Chinese General Social Survey (CGSS) and health resources (Year 2013 and 2015).

	Sample Size	Proportion	Satisfaction Score	Total Health Expenditure as a Percentage of GDP	The Government’s Percentage of Total Expenditure on Healthcare	Out-of-Pocket Percentage of Individuals	Hospital Beds per Thousand Population	Healthcare Workforce per Thousand Population
	(1)	(2)	(3)	(4)	(5)	(6)	(7)	(8)
**2013 and 2015**			Mean	Mean	Mean	Mean	Mean	Mean
Total	15,969	100%	68.5	5.48	29.45	33.12	6.05	2.84
East China (without Shanghai)	3712	23.2%	68.32	4.24	25.83	31.89	5.96	2.97
Central China	3815	23.9%	70.53	5.45	32.84	36.87	5.82	2.36
West China (without Chongqing)	3461	21.7%	70.49	6.57	36.54	32.13	5.87	2.38
Northeast China	2261	14.2%	63.88	5.53	24.51	40.41	6.44	2.67
Beijing	806	5.1%	66.10	7.21	25.43	20.45	7.48	5.60
Shanghai	984	6.2%	64.33	5.59	20.79	20.22	6.89	4.27
Tianjin	511	3.2%	68.84	3.97	25.86	34.20	5.36	3.15
Chongqing	419	2.6%	73.94	5.64	31.23	32.22	4.26	1.58
**2013**								
Total	5566	100%	66.21	5.22	29.59	34.87	5.55	2.74
East China (without Shanghai)	1311	23.6%	67.26	3.94	26.46	33.45	5.2	2.62
Central China	1233	22.2%	66.73	5.26	32.74	39.14	5.97	2.44
West China (without Chongqing)	1174	21.1%	67.01	6.43	37.17	33.77	6.16	2.55
Northeast China	828	14.9%	62.88	5.25	24.5	42.84	5.68	2.46
Beijing	260	4.7%	63.75	6.66	26.9	22.6	5.89	4.77
Shanghai	388	7.0%	62.61	5.41	21.3	19.5	4.68	4.29
Tianjin	204	3.7%	70.60	3.72	25.2	36.4	3.77	2.79
Chongqing	167	3.0%	71.67	5.45	31.5	35.6	4.06	1.62
**2015**								
Total	10,403	100%	69.73	5.62	29.38	32.19	6.31	2.89
East China (without Shanghai)	2400	23.1%	68.91	4.41	25.48	31.04	6.38	3.16
Central China	2582	24.8%	72.35	5.54	32.89	35.78	5.75	2.33
West China (without Chongqing)	2287	22.0%	72.29	6.64	36.22	31.28	5.72	2.29
Northeast China	1434	13.8%	64.46	5.69	24.52	39	6.88	2.78
Beijing	547	5.3%	67.22	7.48	24.73	19.42	8.24	5.99
Shanghai	595	5.7%	65.45	5.71	20.46	20.69	8.34	4.25
Tianjin	307	3.0%	67.66	4.14	26.3	32.73	6.42	3.39
Chongqing	252	2.4%	75.45	5.76	31.05	29.97	4.39	1.55

Note: The total observation number is sample weight-adjusted (N = 15,969). All health resources are equally weighted.

**Table 2:** Grey Relation Degree and Ranking of the Healthcare Resource Factors for Public Satisfaction with Health System in China (Year 2013 and 2015).

2013 and 2015	Total Health Expenditure as a Percentage of GDP	Government’s Percentage of Total Expenditure on Healthcare	Out-of-Pocket Percentage of Individuals	Hospital Beds per Thousand Population	Healthcare Workforce per Thousand Population
Deng ‘s GRA	0.7769	0.82	0.7853	0.7837	0.6865
Rank	4	1	2	3	5
Absolute GRA	0.5904	0.8321	0.6664	0.709	0.696
Rank	5	1	4	2	3
Relative GRA	0.9783	0.9823	0.9802	0.9188	0.8589
Rank	3	1	2	4	5
SDGRA	0.7844	0.9072	0.8233	0.8139	0.7775
Rank	4	1	2	3	5
SSGRA	0.68365	0.82605	0.72585	0.74635	0.69125
Rank	5	1	3	2	4

Note: The total observation number is sample weight-adjusted (N = 15,969). All health resources are equally weighted.

**Table 3:** Grey Relation Degree and Ranking of the Healthcare Resource Factors for Public Satisfaction with Health System in China (Year 2013).

2013	Total Health Expenditure as a Percentage of GDP	Government’s Percentage of Total Expenditure on Healthcare	Out-of-Pocket Percentage of Individuals	Hospital Beds per Thousand Population	Healthcare Workforce per Thousand Population
Deng ‘s GRA	0.745	0.7794	0.7727	0.731	0.6346
Rank	3	1	2	4	5
Absolute GRA	0.6041	0.8521	0.8017	0.6719	0.6967
Rank	5	1	2	4	3
Relative GRA	0.9877	0.9822	0.9975	0.945	0.866
Rank	2	3	1	4	5
SDGRA	0.7959	0.9171	0.8996	0.8084	0.7814
Rank	4	1	2	3	5
SSGRA	0.67455	0.81575	0.7872	0.70145	0.66565
Rank	4	1	2	3	5

Note: The observation number is sample weight-adjusted (N = 5566 in 2013). All health resources are equally weighted.

**Table 4:** Grey Relation Degree and Ranking of the Healthcare Resource Factors for Public Satisfaction with Health System in China (Year 2015).

2015	Total Health Expenditure as a Percentage of GDP	Government’s Percentage of Total Expenditure on Healthcare	Out-of-Pocket Percentage of Individuals	Hospital Beds per Thousand Population	Healthcare Workforce per Thousand Population
Deng ‘s GRA	0.7904	0.8394	0.7836	0.7642	0.6833
Rank	2	1	3	4	5
Absolute GRA	0.5717	0.8005	0.5683	0.7083	0.681
Rank	4	1	5	2	3
Relative GRA	0.9622	0.986	0.9583	0.9151	0.8607
Rank	2	1	3	4	5
SDGRA	0.767	0.8933	0.7633	0.8117	0.7709
Rank	4	1	5	2	3
SSGRA	0.68105	0.81995	0.67595	0.73625	0.68215
Rank	4	1	5	2	3

Note: The observation number is sample weight-adjusted (N = 10,403 in 2015). All health resources are equally weighted.

**Table 5:** Grey Relation Degree and Ranking of the Healthcare Resource Factors for Public Satisfaction with Health System in eight regions of China (Year 2013 and 2015, Deng’s GRA model).

2013 and 2015	Total Health Expenditure as a Percentage of GDP	Government’s Percentage of Total Expenditure on Healthcare	Out-of-Pocket Percentage of Individuals	Hospital Beds per Thousand Population	Healthcare Workforce per Thousand Population
East China (without Shanghai)	0.6391	0.8485	0.9392	0.9796	0.8953
Rank	5	4	2	1	3
Central China	0.8977	0.7401	0.7276	0.8655	0.5995
Rank	1	3	4	2	5
West China (without Chongqing)	0.7261	0.5975	0.9935	0.8819	0.6057
Rank	3	5	1	2	4
Northeast China	0.8647	0.8804	0.5304	0.7522	0.8387
Rank	2	1	5	4	3
Beijing	0.5517	0.8814	0.5722	0.599	0.3333
Rank	4	1	3	2	5
Shanghai	0.8571	0.6782	0.5872	0.6691	0.4922
Rank	1	2	4	3	5
Tianjin	0.5878	0.8373	0.8155	0.7809	0.9959
Rank	5	2	3	4	1
Chongqing	0.8684	0.9167	0.9021	0.5253	0.4178
Rank	3	1	2	4	5

Note: The total observation number is sample weight-adjusted (N = 15,969). All health resources are equally weighted.

## Data Availability

Data available in a publicly accessible repository: (1) CGSS: http://cgss.ruc.edu.cn/; (2) China Public Health Statistical Yearbook 2013&2015.
